# Circulating Tumor Cell Detection by Liquid Biopsy during Early-Stage Endometrial Cancer Surgery: A Pilot Study

**DOI:** 10.3390/biom13030428

**Published:** 2023-02-24

**Authors:** Sarah Francini, Martha Duraes, Gauthier Rathat, Valérie Macioce, Caroline Mollevi, Laurence Pages, Catherine Ferrer, Laure Cayrefourcq, Catherine Alix-Panabières

**Affiliations:** 1 Department of Gynecological and Breast Surgery, Montpellier University Hospital, 34000 Montpellier, France; 2 Clinical Research and Epidemiology Unit, Montpellier University Hospital, 34000 Montpellier, France; 3 Department of Gynecological and Breast Surgery, Nimes University Hospital, 30000 Nimes, France; 4 Laboratory of Rare Human Circulating Cells, University Medical Center of Montpellier, 34000 Montpellier, France; 5 CREEC, MIVEGEC, University of Montpellier, CNRS, IRD, 34000 Montpellier, France; 6 European Liquid Biopsy Society (ELBS), 20246 Hamburg, Germany

**Keywords:** endometrial cancer, liquid biopsy, circulating tumor cells, laparoscopy

## Abstract

The recurrence of non-metastatic endometrial carcinoma (EC) (6 to 21%) might be due to disseminated tumor cells. This feasibility study investigated whether circulating tumor cells (CTCs) were detectable in blood samples from the peripheral and ovarian veins of 10 patients undergoing laparoscopic resection of stage I-II EC between July 2019 and September 2021. CTCs were detected using the CellSearch^®^ system (i) preoperatively (T0) in peripheral blood, (ii) after ovary suspensory ligament pediculation in ovarian vein blood (T1), and (iii) before colpotomy in peripheral blood (T2). CTCs were detected only in ovarian vein samples in 8/10 patients. The CTC median number did not differ with patient age (37 (min-max: 0–91) in <70-year-old vs. 11 (0–65) in ≥70 year-old women, *p* = 0.59), tumor grade (15 (0–72) for grade 1 vs. 15 (0–91) for grade 2, *p* = 0.97), FIGO stage (72 (27–91) vs. 2 (0–65) vs. 3 (0–6]) for stage IA, B, and II, respectively; *p* = 0.08), and tumor size (40 (2–72) for size < 30 mm vs. 4 (0–91) for size ≥ 30 mm, *p* = 0.39). Estrogen receptor-positive CTCs and CTC clusters were identified. The prognostic and therapeutic values of CTCs released during EC surgery need to be determined.

## 1. Introduction

Endometrial carcinoma (EC) is the most common gynecological cancer in Europe, with a 5-year prevalence of 34.7% (https://gco.iarc.fr/today/data/factsheets/cancers/24-Corpus-uteri-fact-sheet.pdf; accessed on December 2020). In 2018, the estimated number of new EC cases was 121,578 and the number of related deaths was 29,638 in Europe. Incidence has been rising due to population aging and increased obesity rate (https://gco.iarc.fr/today/data/factsheets/cancers/24-Corpus-uteri-fact-sheet.pdf; accessed on December 2020). In France, the National Cancer Institute reported a 5-year survival rate of 74% for women with EC diagnosed in 2010–2015 (https://www.santepubliquefrance.fr/docs/survie-des-personnes-atteintes-de-cancer-en-france-metropolitaine-1989-2018-corps-de-l-uterus-:~:text=Accueildocs-,Survie des personnes atteintes de cancer en France métropolitaine, 2018-Corps de l’utérus&text=En France métropolitaine, pour l,l’utérus de 2 415; accessed on December 2020).

Lymph node status plays an important role in the outcome prediction of patients with operable EC. The risk of recurrence of non-metastatic EC is significant, from 6 to 21% [1]. It is thought that EC recurrences are due to occult disseminated tumor cells that are not detected or removed surgically [2]. These early disseminated tumor cells cannot be detected through the quantification of conventional serum tumor markers or by imaging. Therefore, it is crucial to develop techniques to improve the assessment of the response to treatment and risk of recurrence in these early-stage cancers.

Hematogenous dissemination during tumor mobilization has been demonstrated during gynecological cancer surgery [3,4]. Laparoscopy has become the standard surgical procedure for gynecological cancers. However, controversies remain regarding its potential harmful effects on tumor dissemination. It has been suggested that laparoscopic approaches might further promote tumor cell detachment and mobilization. A randomized trial showed that cervical cancer prognosis was poorer in patients managed by laparoscopy than laparotomy [4]. Since this trial, surgical teams have stopped using the uterine manipulator during gynecological cancer resection by laparoscopy, due to an increased risk of tumor mobilization. The uterine manipulator is an instrument that can be inserted into the uterus at the beginning of intervention. It helps to expose the pelvis by moving the uterus in different directions to identify structures and find anatomical landmarks such as vaginal fornices for colpotomy. This issue might also concern laparoscopy of EC, and should be evaluated.

In the last 10 years, many clinical oncology researchers have been interested in the detection and characterization of circulating tumor cells (CTCs) [5]. Numerous clinical studies and meta-analyses that included large cohorts of patients have demonstrated that CTC count is an important prognostic factor and a predictive factor of response to treatment in metastatic breast, prostate, and colorectal cancer [6,7,8,9]. It may also be used to guide postoperative treatment. CTC clinical validity and clinical utility have been shown in breast cancer [9,10]. When added to clinicopathological predictive models, CTC count (but not serum tumor markers) improves the prognostication of metastatic breast cancer [9]. Furthermore, the identification of one or more CTCs also predicts worse outcome in patients with non-metastatic cancer [11]. Similarly, during systemic therapy for metastatic disease, an increase in CTC count at any time indicates rapid disease progression [12]. 

However, CTC detection and prognostic impact have been poorly studied in EC [3,6,13], and their role in this cancer remains unclear. CTCs have been detected in blood samples of patients with EC using different detection assays, such as cytokeratin-20 mRNA amplification [14], the CELLection™ Epithelial Enrich kit (Invitrogen, Dynal, Oslo, Norway) [15], and the CellSearch (Menarini—Silicon Biosystems, Bologna, Italy), and the positive cut-off level varied among studies. Indeed, all patients with recurrent disease were CTC-positive in one study [14], but not in the other study [6]. Far less is known about CTCs in patients with early-stage and stage II EC. Bogani et al. evaluated CTC detection in 28 patients with grade 3 EC. They detected CTCs mainly in patients with stage III and IV EC. Among patients with recurrence, none had CTCs [6]. Lemech et al. detected CTCs in 60% of patients with metastatic disease (total *n* = 30) [3]. Most of these patients had grade 3 EC. Ni et al. evaluated CTC detection in 40 patients with stage I–IV and grade 1–3 EC. They could detect CTCs in 15% of patients, but did not find any correlation with stage or grade [6,13], and the positive cut-off level varied among studies. Indeed, all patients with recurrent disease were CTC-positive in one study [14], but not in the other study [6]. Far less is known about CTCs in patients with early-stage and stage II EC. Bogani et al. evaluated CTC detection in 28 patients with grade 3 EC. They detected CTCs mainly in patients with stage III and IV EC. Among patients with recurrence, none had CTCs [6]. Lemech et al. detected CTCs in 60% of patients with metastatic disease (total *n* = 30) [3]. Most of these patients had grade 3 EC. Ni et al. evaluated CTC detection in 40 patients with stage I–IV and grade 1–3 EC. They could detect CTCs in 15% of patients, but did not find any correlation with stage or grade [13].

Most studies have focused on CTC detection and enumeration in peripheral blood [6,7,8,9]. Fewer reports have tested the hypothesis that the chance of capturing and detecting CTCs might be higher in vessels closer to the tumor, especially in the main veins that drain blood from the organ invaded by the cancer. However, it is well known that tumor-proximal liquid biopsy improves CTC diagnostic performance because sampling blood from a tumor draining vein greatly increases the chances of detecting CTCs directly released by the tumor [16], particularly for some cancer types. Indeed, unlike breast and prostate tumors, CTC release in the peripheral blood by colorectal cancer is a rare event, and, consequently, their detection is difficult in clinical practice [17]. Jiao et al. found that the median CTC number before surgery was higher in the portal circulation and hepatic vein than in peripheral blood [18]. Another study compared CTC identification in peripheral and portal vein blood samples in 41 patients undergoing upfront surgery for pancreatic ductal adenocarcinoma [19]. CTCs were detected (CellSearch^®^) in 39% of peripheral and 58.5% of portal vein blood samples. CTC presence in the portal blood was a predictive factor of liver metastasis. Our hypothesis was that in early-stage EC, CTCs may not be detectable in peripheral blood and that the chances of detection might be higher close to the tumor, particularly using blood sampled from the ovarian vein.

Therefore, the aim of this study was to determine whether CTCs that express or do not express estrogen receptor (ER) can be detected in blood sampled from ovarian and peripheral veins during laparoscopic resection of stage I-II EC. Then, the correlation of the detected CTCs with clinicopathologic characteristics was analyzed.

## 2. Materials and Methods

### 2.1. Patient Recruitment

Ten patients with primary stage I-II EC who underwent laparoscopic surgery at the departments of gynecological surgery of the Montpellier and Nimes University Hospitals between July 2019 and September 2021 (ClinicalTrials.gov accessed on 12 December 2020 Identifier: NCT04021459) were recruited. All patients underwent pelvic MRI and only patients with stage FIGO I and II EC were included. Patients with non-endometrioid carcinoma, laparotomy, other concomitant cancer, and preoperative hemoglobin levels <9 g/L were excluded. The present study was approved by the local Ethics Committee (IDRCB 2019-A01105-52) and a signed informed consent was obtained from all patients with EC.

### 2.2. Surgical Procedure

Surgery was performed by gynecological surgeons with curative intent. For laparoscopy, pneumoperitoneum was established with CO_2_ and intra-abdominal pressure ≤ 15 mmHg. Abdominal exploration was carried out to exclude extended disease. Peritoneal cytology was systematically carried out. If indicated, pelvic lymph nodes were dissected before hysterectomy. All procedures were performed without uterine manipulator.

### 2.3. Blood Sampling

**Figure 1:**
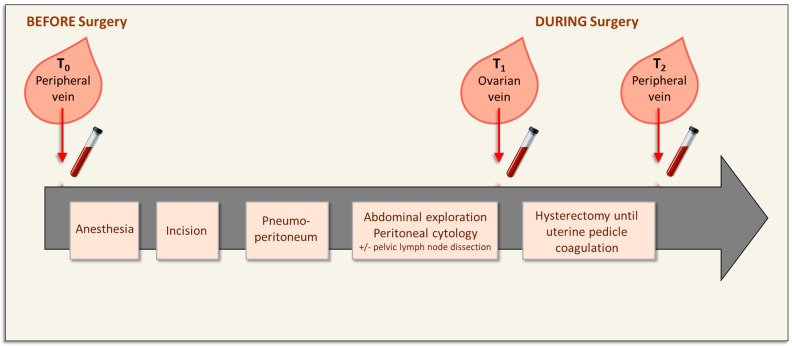
**Blood collection before and during surgery of early-stage endometrial cancer**. The initial blood sample (T_0_) was collected from a peripheral vein just before anesthesia and surgery. The other blood samples were obtained during surgery: T_1_ (ovarian vein) after pediculation of the ovary suspensory ligament, and T_2_ (peripheral vein) after uterine pedicle coagulation.

Peripheral whole blood samples (10 mL) were collected using an intravenous cannula before entering the operating room (T_0_), when the peripheral venous catheter was put in place, and then after section of the uterine pedicles before colpotomy (T_2_) (Figure 1). Intraoperative sampling (T_1_) from the right and/or left ovarian vein(s) was performed using an oocyte puncture needle (Cook^®^, 330 mm, 17 G), inserted through a 5- or 10-mm laparoscopic trocar and connected to a 5 mL syringe. The catheter was pre-purged with heparinized serum. At the beginning, an epicranial needle, inserted through a 5 mm laparoscopic trocar, was tested for ovarian vein sampling, but this method was technically difficult and was not retained. The T_1_ sample was collected after lymph node dissection (if performed) and pediculation of the ovary suspensory ligament (Figure 1).

Blood samples were drawn into tubes (one per time point) that contained a cell preservative (CellSave Preservative Tubes, #7900005, Menarini—Silicon Biosystems, Bologna, Italy). Blood samples were sent, at room temperature, to the Laboratory of Rare Human Circulating Cells (LCCRH), Montpellier University Medical Center, France.

### 2.4. CTC Enumeration and ER Expression Detection

Blood samples were analyzed within 96 h after reception using the CellSearch^®^ system and the CXC Kit (#7900017, Menarini—Silicon Biosystems, Bologna, Italy) according to the manufacturer’s recommendations. Briefly, epithelial cells were captured with an anti-epithelial cell adhesion molecule (EpCAM) antibody and detected with anti-cytokeratin (CK) 8, 18, and 19 antibodies. An anti-CD45 antibody was used to identify and exclude leukocytes. Nuclei were counterstained with 4,6-diamidino-2-phenylindole (DAPI). After enrichment and immunocytochemical staining, immunomagnetically labeled cells were kept in a strong magnetic field and scanned. Experienced researchers interpreted the results of these analyses. A blood sample of 7.5 mL was considered positive when at least 1 CK^(+)^, DAPI^(+)^, CD45^(−)^ cell was detected (5). This automated system allows the addition of a supplemental marker in the fourth channel to phenotypically characterize CTCs; in this pilot study, an anti-ER antibody (Menarini—Silicon Biosystems, Bologna, Italy) was added to detect ER^(+)^ CTCs.

### 2.5. Statistical Analysis

A number of observations and percentages were reported for qualitative variables; median, minimum, and maximum values were presented for quantitative variables. Continuous clinicopathologic variables were dichotomized at the median value. CTC count (mean number per ml) was compared with the clinicopathologic characteristics using the Wilcoxon Mann–Whitney test or the Kruskal–Wallis test. All statistical tests were two-sided and *p* < 0.05 was considered significant. Statistical analyses were performed with SAS version 9 (SAS Institute, Cary, NC, USA).

## 3. Results

### 3.1. Patient Cohort

**Figure 2:**
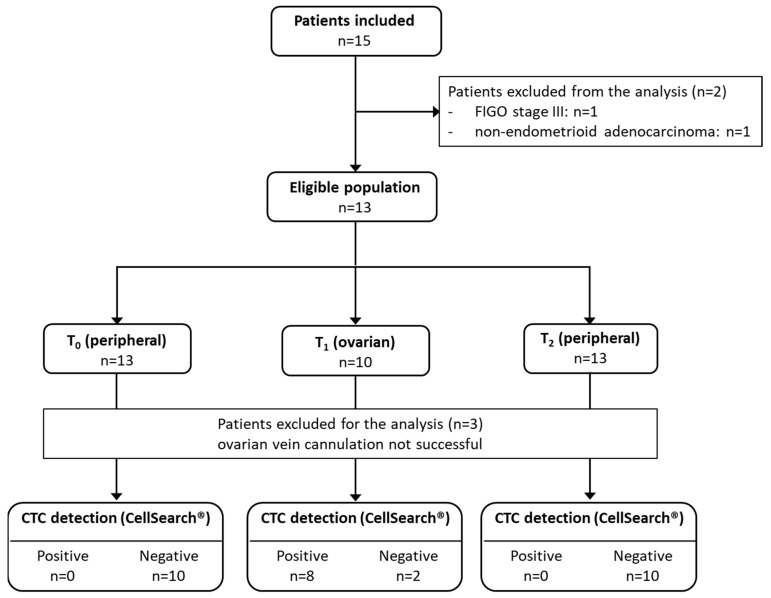
Flowchart showing the number of included and evaluable patients.

During the study period, 31 patients were screened and 15 patients were included, among whom 5 were excluded due to the detection of carcinosis during abdominal exploration (*n* = 1), unsuccessful cannulation of the ovarian vein using an epicranial needle (see Methods) (*n* = 3), and non-endometrioid adenocarcinoma at histological evaluation (*n* = 1) (Figure 2).

Ten patients remained for the final analysis. Their clinicopathologic characteristics are summarized in Table 1.

### 3.2. Surgical Procedure and Blood Collection

Pelvic lymph nodes were dissected in eight patients (*n* = 6 sentinel lymph node biopsies). The median intervention duration was 153 (min; max: 90; 300) minutes. The median time between pneumoperitoneum and T_1_ was 43 (min; max: 10; 90) minutes. The median time between T_1_ and T_2_ was 10 (min; max: 3; 30) minutes. The total blood volume needed for CTC detection/analysis (7.5 mL) could be collected during peripheral vein sampling; conversely, the median volume collected during ovarian vein sampling was 1.5 (min; max: 0.5; 3.8). No complications were reported during blood sampling.

### 3.3. CTC Enumeration before, during, and after Surgery and ER Expression

**Figure 3:**
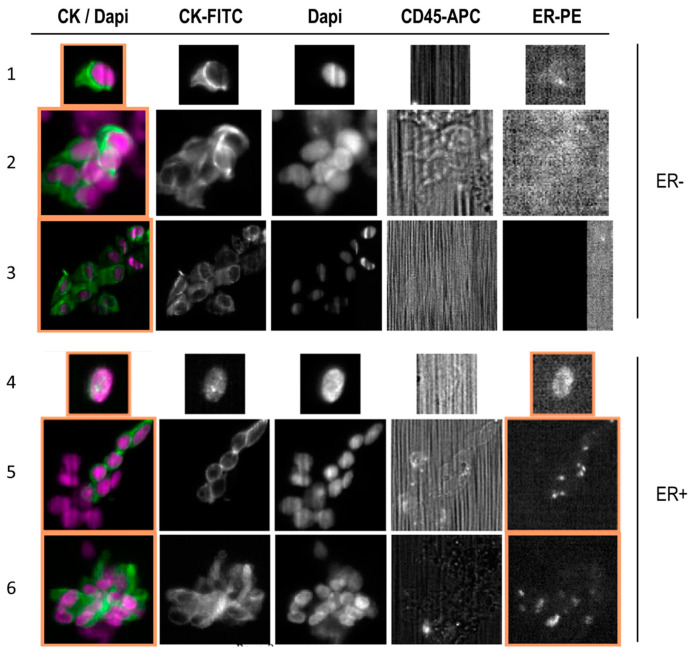
**Representative images of circulating endometrial cancer cells detected with the CellSearch^®^ system in ovarian vein blood samples from patients with early-stage endometrial cancer during surgery**. CTCs were identified as EpCAM^(+)^, CK^(+)^, DAPI^(+)^, and CD45^(−)^ cells. In the fourth channel, an antibody against ER was added to characterize CTCs. Individual ER^(−)^ CTCs (line 1), clusters of ER^(−)^ CTCs (lines 2–3), individual ER^(+)^ CTCs (line 4), and clusters of ER^(+)^ CTCs (lines 5–6) were observed in the ovarian vein blood samples of patients with early-stage endometrial cancer. Abbreviations: EpCAM, epithelial cell adhesion molecule; CK, cytokeratin; DAPI, 4′,6-diamidino-2-phenylindole; FITC, fluorescein isothiocyanate; APC, allophycocyanin.

CTCs were not detected in the peripheral blood samples (T_0_ and T_2_). Conversely, they were identified in the ovarian vein samples (T_1_) of 8/10 patients (median CTC count: 11/mL; range: 2–91/mL of blood). For the two patients without CTCs in the ovarian vein, one had a small blood sample volume (0.5 mL) and the other had a clot in the sample. Among the eight CTC-positive ovarian vein samples, five (62%) included ER^(+)^ CTCs (Figure 3). The median number of ER^(+)^ CTCs was 6 (range 1–23).

The CellSearch^®^ system also allowed the detection of single CTCs and CTC clusters in ovarian vein samples (Figure 3). CTC clusters were detected in four out of eight patients (50%). The number of CTCs in clusters ranged from 2 to >20 cells. However, as the number of cells was difficult to evaluate in big clusters (>10 cells), CTCs in clusters were counted as a single event. The ER status of clusters was not homogenous. In two patients, both ER^(+)^ and ER^(−)^ clusters were detected, whereas the other two patients had only ER^(−)^ clusters.

No correlation was found between clinicopathological features and the median number of CTCs per ml of blood from ovarian vein samples (Table 2).

## 4. Discussion

The purpose of this observational pilot study was to determine whether CTC could be detected in peripheral and ovarian blood samples collected during laparoscopic resection of early-stage EC. We demonstrated that CTC can be detected in ovarian vein blood samples. This finding now leads to the question of whether CTC enumeration could be a useful biomarker for assessing recurrence risk.

In the literature, the CTC detection rate in the peripheral blood of patients with EC ranges from 7% to 75% [3,6,7,13,20]. This variability may be due to differences in population characteristics, CTC detection techniques, and number of patients studied. Here, peripheral blood samples were analyzed using the CellSearch^®^ system, currently the only FDA-cleared technique for CTC detection in metastatic breast, prostate and colon cancers [5]. Its effectiveness has been demonstrated by several studies, including in EC [3,6,9,10,13,16]. Furthermore, the CTC detection rate in peripheral blood samples increases in high-grade and advanced FIGO stage EC [3,6]. In the present study, only patients with early-stage and grade 1 and 2 EC were included, thus explaining the absence of CTC detection in peripheral blood before surgery (T_0_). Conversely, CTCs were found in the ovarian vein blood sample of 80% of patients (T_1_), but not in the peripheral blood sample collected at T_2_. Blood sampling close to the tumor during surgery increases CTC detection rate and also the number of detected CTCs [16]. Denève et al., Buscail et al., and Dong et al. also found higher rate of CTCs close to the tumor in patients treated for colorectal, pancreatic, and lung cancer, respectively [21,22,23]. In summary, blood sampling close to the tumor, in this case the ovarian vein, will allow us to monitor CTC count in patients with early-stage EC and to evaluate the correlation between CTC count and the risk of recurrence in a larger cohort.

None of the peripheral blood samples collected at T_2_ was positive, although they were collected only 10 min (mean) after T_1_ (ovarian vein sample). As CTC half-life is 1–2.4 h [24], we could hypothesize that CTCs released from the EC hardly reached the peripheral circulation because they were trapped in other organs. This could explain the difficulties of detecting CTCs in peripheral blood samples from patients with EC.

The CellSearch^®^ system also allowed the addition of an anti-ER antibody that labeled CTCs in five out of eight patients. ER expression is a prognostic biomarker in EC, and the loss of this receptor (in association with progesterone receptors) is associated with unfavorable outcomes [25]. Thus, it could be interesting to investigate the ER status of CTCs released by the tumor during surgery to assess whether the number of ER^(+)^ CTCs might help clinicians to discriminate patients at high and low risk of recurrence. Furthermore, CTC clusters were detected in four out of eight CTC-positive ovarian vein blood samples. CTC clusters are more resistant and have greater metastatic potential than single CTCs [26,27]. Wang C. et al. reported that the presence of CTC clusters is associated with poor progression-free survival and overall survival in metastatic breast cancer [28]. Thus, their evaluation during surgery may help to identify patients at higher risk of recurrence.

The absence of CTCs in two ovarian vein blood samples could be a false negative result due to the difficulty of analysis (low blood volume for one sample, and presence of a clot for the other). For this reason, and also because of the limited number of samples, clinicopathological features were not compared with CTC presence/absence in the ovarian vein sample. Moreover, the median CTC count in the ovarian vein was not different in patients divided according to their age, tumor grade, FIGO stage, and size. This might be explained by the small sample size. However, CTC presence in the ovarian vein might mostly reflect the surgery’s contribution to the mechanical release of tumor ovarian cells into the blood circulation, which is independent of clinical characteristics. Bogani et al. found that deep myometrial invasion (>50%) was an independent predictor of CTC presence [6]. This is consistent with the study by Mariani et al. showing that deep myometrial invasion is a risk factor of metastatic recurrence [29]. Previous studies have found that CTC presence in peripheral blood is associated with tumor size > 5 cm [3] and cervix extension [6], whereas other studies did not detect any association between CTC presence and tumor grade, depth of myometrial invasion, or metastatic disease [13,20].

Few studies evaluated the link between CTC detection and recurrence and survival [14,30,31,32]. Sawabata et al. [32] found that in patients without preoperative CTCs, CTC detection immediately after non-small cell lung cancer resection was associated with tumor vessel invasion, lymphovascular invasion, and pleural invasion. Distant metastases were more common in patients with CTC clusters detected after surgery. Moreover, the 2-year recurrence-free survival rate was higher in patients without CTC (94.6%) vs. one CTC (62.5%) and CTC clusters (52.9%) [32]. Wind et al. showed that in patients undergoing (laparoscopic or open) surgery for primary colon cancer [33], the CTC count significantly increased intra-operatively and was significantly higher in portal blood than in peripheral blood samples. Ramirez et al. found that prognosis was worse in patients treated for cervical cancer by laparotomy vs. laparoscopy [4]. One of the explanations was that laparoscopic approaches might enhance tumor cell detachment and mobilization. However, other studies showed that laparoscopy does not increase CTC release compared with open surgery [33,34,35,36].

In our study, CTCs were detected in the ovarian vein blood sample collected during laparoscopy. However, the CTC status could not be correlated with recurrence or survival, which were not assessed. Two studies [6,13] analyzed the link between CTCs and survival in EC, but only in patients with higher-stage cancers, and they did not evaluate the impact of surgery. Specifically, Ni et al. followed 40 patients for 13 months [13] and reported only one recurrence in a CTC^(−)^ patient. Bogani et al. found that 21% (6/28) of patients with high-risk EC had a recurrence [6], all with CTC^(−)^ blood samples. Many studies found a relationship between CTC presence and survival in other cancer types. For instance, Cohen et al. showed that CTC number is an independent predictor of progression-free survival and overall survival in patients with metastatic colorectal cancer (*n* = 430 patients) [31]. De Bono et al. found a relationship between post-treatment CTC count and overall survival in 276 patients with castration-resistant prostate cancer [30].

To our knowledge, this is the first study to report the perioperative rate of CTCs in patients undergoing laparoscopic surgery for EC. The presence and count of CTCs during surgery, and the evaluation of their ER status, could help to predict the clinical outcome of these patients. Two limitations of this study were the small sample size, and CTC detection only in ovarian vein samples and not in peripheral vein blood samples during surgery. Therefore, these preliminary data must be validated in a larger cohort of patients with early-stage EC. Moreover, to increase the chance of detecting CTCs, higher volumes of peripheral blood (e.g., 20–30 mL) will be used.

## 5. Conclusions

This study confirmed the feasibility of CTC detection in blood samples from the ovarian vein of patients undergoing laparoscopic surgery for early-stage EC. CTCs were detected in the ovarian vein blood sample of 80% of patients, but not in the peripheral blood samples. In addition, ER^(+)^ CTCs and CTC clusters were observed in ovarian vein samples, and this might be of prognostic interest. The CTC number was not correlated with clinicopathologic characteristics. However, this was a pilot study, and these preliminary data must be validated in a larger cohort of patients with early-stage EC and using larger volumes of peripheral blood (e.g., 20–30 mL). Lastly, the prognostic and potential therapeutic features of CTC identification during laparoscopy in patients with EC must be investigated. A key point will be to assess the relationship between CTCs and/or presence of ER^(+)^ CTC/CTC clusters and the clinical outcome using liquid biopsy samples collected close to the tumor.

## Figures and Tables

**Table 1:** Patients’ characteristics (*n* = 10).

Characteristics	Values
Age, median (min; max)	70 (46; 86)
FIGO stage, N (%)	
IA	5 (50)
IB	5 (50)
Biopsy grade, N (%)	
1	5 (50)
2	5 (50)
Tumor size (mm), median (min; max)	28 (8; 37)
Pelvic lymph node dissection, N (%)	
No	2 (20)
Sentinel lymph node biopsy	6 (60)
Pelvic lymphadenectomy	2 (20)

Abbreviations: SD, standard deviation; FIGO, Federation of Gynecology and Obstetrics.

**Table 2:** CTC count (median number per ml of blood) in ovarian vein samples with the patients’ clinicopathological features.

Characteristics	N	Median (Min–Max)	*p*
Age			0.59
<70 years	4	37 (0–91)	
≥70 years	6	11 (0–65)	
FIGO stage			0.08
IA	3	72 (27–91)	
IB	5	2 (0–65)	
II	2	3 (0–6)	
Biopsy grade			0.97
1	4	15 (0–72)	
2	5	15 (0–91)	
3	1	6 (-)	
Tumor size (mm)			
<30mm	4	40 (2–72)	0.39
30mm	6	4 (0–91)	

Abbreviations: FIGO, Federation of Gynecology and Obstetrics.

## Data Availability

Not applicable.
